# Pyroptosis mediates osteoporosis via the inflammation immune microenvironment

**DOI:** 10.3389/fimmu.2024.1371463

**Published:** 2024-06-03

**Authors:** Te Chen, Linyu Jin, Jingyi Li, Yikai Liu

**Affiliations:** ^1^ Division of Joint Surgery, Department of Orthopaedics, Hainan General Hospital, Hainan Affiliated Hospital of Hainan Medical University, Haikou, Hainan, China; ^2^ Department of Orthopedics, Shanghai Key Laboratory for Prevention and Treatment of Bone and Joint Diseases, Shanghai Institute of Traumatology and Orthopedics, Ruijin Hospital, Shanghai Jiaotong University School of Medicine, Shanghai, China; ^3^ Department of Endocrinology, Hainan General Hospital, Hainan Affiliated Hospital of Hainan Medical University, Haikou, Hainan, China

**Keywords:** pyroptosis, osteoporosis, inflammatory immune microenvironment, inflammatory factor, bone metabolism

## Abstract

Osteoporosis represents a systemic imbalance in bone metabolism, augmenting the susceptibility to fractures among patients and emerging as a notable mortality determinant in the elderly population. It has evolved into a worldwide concern impacting the physical well-being of the elderly, imposing a substantial burden on both human society and the economy. Presently, the precise pathogenesis of osteoporosis remains inadequately characterized and necessitates further exploration. The advancement of osteoporosis is typically linked to the initiation of an inflammatory response. Cells in an inflammatory environment can cause inflammatory death including pyroptosis. Pyroptosis is a recently identified form of programmed cell death with inflammatory properties, mediated by the caspase and gasdermin families. It is regarded as the most inflammatory form of cell death in contemporary medical research. Under the influence of diverse cytokines, macrophages, and other immune cells may undergo pyroptosis, releasing inflammatory factors, such as IL-1β and IL-18. Numerous lines of evidence highlight the pivotal role of pyroptosis in the pathogenesis of inflammatory diseases, including cancer, intestinal disorders, hepatic conditions, and cutaneous ailments. Osteoporosis progression is frequently associated with inflammation; hence, pyroptosis may also play a role in the pathogenesis of osteoporosis to a certain extent, making it a potential target for treatment. This paper has provided a comprehensive summary of pertinent research concerning pyroptosis and its impact on osteoporosis. The notion proposing that pyroptosis mediates osteoporosis via the inflammatory immune microenvironment is advanced, and we subsequently investigate potential targets for treating osteoporosis through the modulation of pyroptosis.

## 1 Introduction

Osteoporosis is a chronic metabolic disorder influenced by various genetic factors, resulting in diminished bone mineral density and deterioration of the local bone microarchitecture. This condition predisposes individuals to heightened bone fragility and an elevated risk of fractures (1). Statistics reveal that one-third of women and one-fifth of men in the global elderly population are afflicted by osteoporosis (2). Consequently, osteoporosis is a substantial factor contributing to both morbidity and mortality in the elderly population.

Osteoporosis is categorized into primary forms, encompassing postmenopausal osteoporosis (PMOP), senile osteoporosis (SOP), and idiopathic osteoporosis (IOP), as well as secondary osteoporosis. It is crucial to emphasize that primary osteoporosis represents the predominant manifestation. Primary osteoporosis is commonly linked to aging and estrogen deficiency, with a higher prevalence observed in individuals aged 50 and above (3). Secondary osteoporosis arises from non-specific diseases and medications (e.g., glucocorticoids) that adversely affect the body’s bone metabolism (4). The homeostasis of bone tissue hinges on the delicate balance of bone metabolism. Ordinarily, there is a relative equilibrium between osteoblasts’ osteogenesis and osteoclasts’ osteoclastic activity; when the factors causing bone loss outweigh bone synthesis, a decline in bone density ensues, culminating in osteoporosis (5). However, the precise mechanism of osteoporosis remains inadequately understood, underscoring the imperative for in-depth exploration into the pathogenesis of osteoporosis and the identification of potential therapeutic targets aimed at ameliorating osteoporotic bone loss to mitigate the risk of fractures.

Pyroptosis, a programmed cell death modulated by inflammasomes, is intricately tied to inflammation. It has been documented in diverse inflammatory conditions (6). Inflammation frequently contributes to the onset of osteoporosis (7). The process of pyroptosis is usually associated with osteopenia and may not be directly involved in the process of osteoporosis, but rather may regulate bone metabolism indirectly by interacting with immune cells and other related cells. Pyroptosis may manifest in immune cells and bone metabolic cells within the bone microenvironment, influencing bone metabolism through the secretion of inflammatory factors, thereby establishing an inflammatory immune microenvironment and, consequently, culminating in osteoporosis. This article succinctly reviews recent studies on the involvement of pyroptosis in the pathogenesis of osteoporosis, aiming to delve deeper into the role of pyroptosis in osteoporosis and offering the prospect of novel insights and inspiration for readers.

## 2 The process of osteoporosis is concomitant with inflammation

### 2.1 Osteoporosis is intricately associated with estrogen deficiency

Osteoporosis is distinguished by a perturbation in bone metabolism. As per the World Health Organization (WHO), osteoporosis is defined by a bone mineral density (BMD) score equal to or less than -2.5 at the femoral neck or lumbar spine. This condition is linked to heightened bone metabolism, elevating the susceptibility to fractures (8). The preservation of bone tissue homeostasis hinges on the continual generation of new cells and the controlled demise of old cells. Typical bone metabolism and remodeling sustain a dynamic equilibrium within the human body. Osteoblasts, derived from bone marrow mesenchymal stem cells (BMSCs), engage in ongoing protein and calcium synthesis to facilitate new bone formation. In contrast, multinucleated osteoclasts, originating from the mononuclear cell lineage of hematopoietic stem cells, undergo differentiation and fusion to resorb aged bone (9). Osteoporosis ensues when bone resorption outpaces bone formation. With increasing age, bone metabolism-related cell regeneration is diminished, accompanied by the onset of cell senescence. Bone marrow mesenchymal stem cells have a reduced ability to differentiate and proliferate due to senescence, which can result in reduced osteogenesis and the onset of senile osteoporosis. Estrogen, vital for maintaining endocrine, cardiovascular, and skeletal homeostasis in the human body, plays a pivotal role (10). Insufficient estrogen results in bone loss in both cortical and cancellous bone, significantly elevating the risk of osteoporosis in menopausal women (11). Estrogen binds to its receptor situated within the endoplasmic reticulum, proceeding to translocate into the nucleus through endoplasmic reticulum dimerization to interact with DNA sequences (12). In estrogen deficiency, the normal cycle of bone metabolism is disrupted, a process that may be due to the presence of estrogen receptors in osteoblasts. In an oestrogen-deficient environment, the inhibition of osteoclast proliferation and activation is reduced, and when normal osteoblast activity is reduced, osteoclast resorptive activity is increased, leading to osteoporosis (13). Inflammatory factors, including IL-1, IL-6, IL-17, and TNF-α, alongside osteoprotegerin (OPG) produced by T lymphocytes, B lymphocytes, macrophages, and dendritic cells, are included among the target genes of the estrogen receptor (7). IL-10 knockout mice develop osteoporosis and have significantly increased expression of the inflammatory factors IL-6 and TNF in bone, and treatment with E2 ameliorates this process, suggesting that the development of inflammation in bone is closely linked to estrogen deficiency (14). Estrogen inhibits osteoclast differentiation by impeding RANKL/M-CSF-induced activator protein 1 (AP-1) transcription (15). Estrogen suppresses RANKL-induced osteoclast differentiation in human monocytes by inhibiting nuclear factor kappa-B (NF-κB) phosphorylation through estrogen receptor-α (Erα) binding to the scaffolding protein breast cancer anti-estrogen resistance protein-1 (BCAR1) (16). Estrogen diminishes apoptosis in differentiated osteoblasts by inducing autophagy, thus extending their lifespan and fostering osteogenesis (17). E Estrogen is also capable of upregulating bone morphogenetic protein 4 (BMP-4)-induced phosphorylation of SMAD1/5/8 in MC3T3-E1 osteoblasts, thereby fostering osteogenesis (18). ERα in Osterix1-expressing osteoprogenitors promotes osteoblast proliferation and differentiation by augmenting Wnt/β-catenin signaling (19). Estrogen deficiency modifies the composition of the bone matrix, rendering it more fragile (20). Estrogen also impacts the metabolism of other hormones, augmenting the secretion of calcitonin, a hormone that hinders bone resorption (21). Estradiol enhances bone formation by activating parathyroid cells, leading to the stimulation of parathyroid hormone (PTH) expression (22). Therefore, the progression of osteoporosis is intricately intertwined with estrogen deficiency.

### 2.2 The influence of the inflammatory immune microenvironment on bone metabolism

**Figure 1:**
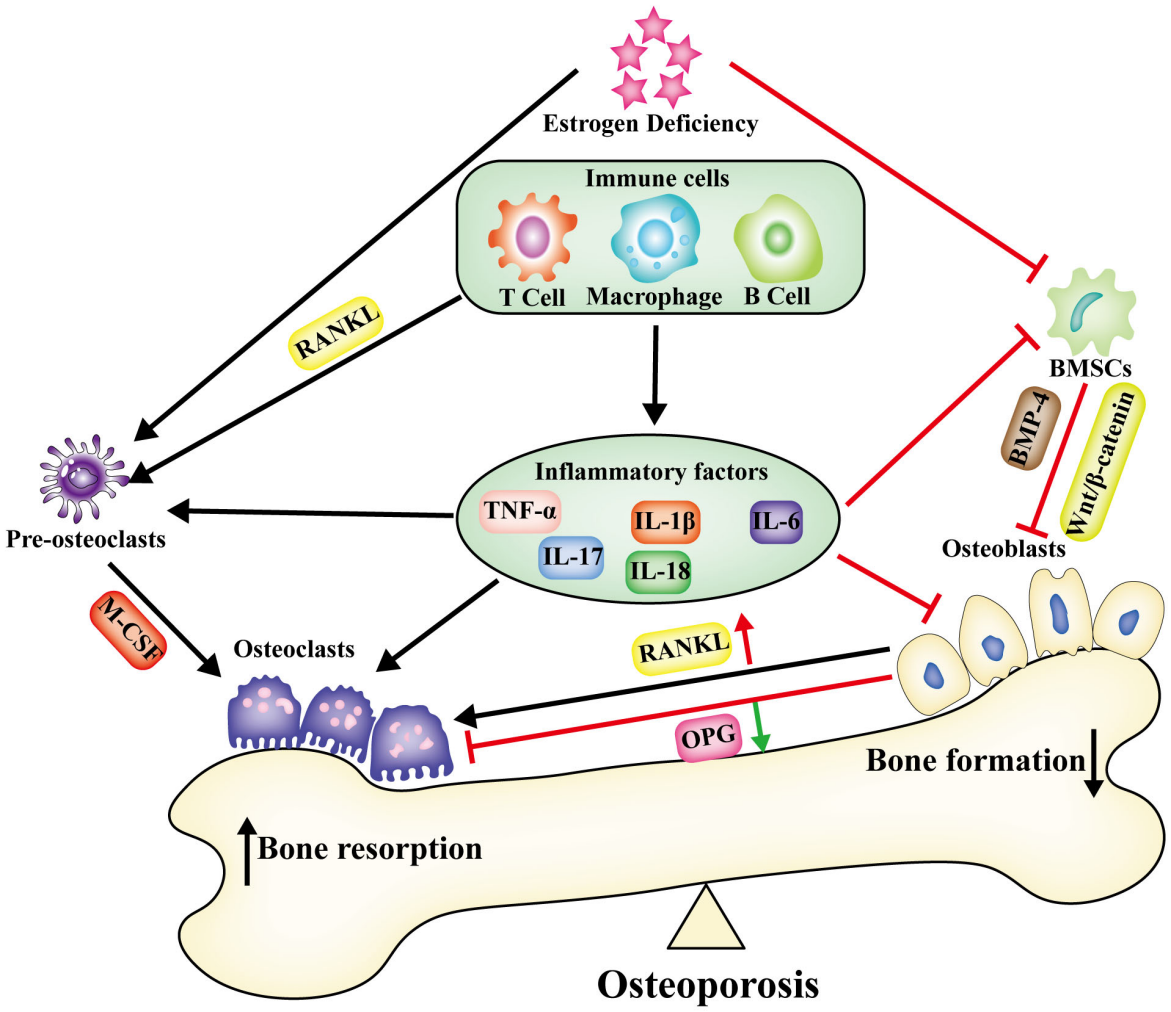
Under the condition of estrogen deficiency, alterations occur in the immune microenvironment within bone tissue. Immune cells, together with the inflammatory factors they release, establish an enduring chronic inflammatory microenvironment. This disturbance in bone metabolism disrupts the equilibrium, leading to a compounded effect of increased bone resorption and reduced bone synthesis, ultimately culminating in osteoporosis.

The immune environment is intricately connected to the regulation of bone metabolism and encompasses immune cells along with the immune factors they release. Human immune cells, comprising T lymphocytes, B lymphocytes, macrophages, and dendritic cells, are subject to the influence of estrogen (23). T and B cells secreting RANKL escalate in cases of human estrogen deficiency. Conditions of estradiol deficiency result in prolonged production of the pro-inflammatory cytokines TNF-α and IL-17, culminating in the establishment of a chronic inflammatory microenvironment (24). The expression of numerous inflammatory factors (IL-1β, IL-6, IL-17, and TNF-α) markedly increased during the progression of osteoporosis. This set of inflammatory factors induced osteoblasts to enhance RANKL expression, fostering osteoclast maturation and subsequent bone resorption (25). Analysis of bone tissue from ovariectomized (OVX) mice unveiled a notable elevation in the expression of pyroptosis-related inflammatory factors IL-1β, IL-18, and TNF-α. Conversely, the expression levels of these inflammatory factors were markedly diminished in NLRP3 knockout OVX mice (26). Under LPS stimulation, mouse macrophages increased secretion of IL-1β and IL-6, creating a pro-inflammatory immune microenvironment (27). Osteoarthritis is a chronic disease that involves inflammation. The expression of IL-1β is significantly increased in the joints of patients with OA, indicating that IL-1β plays a crucial role in creating the inflammatory immune microenvironment (28). However, the number of osteoclasts in the bone marrow cavity of IL-1 receptor knockout mice was significantly reduced. This indicates that the immune-inflammatory microenvironment created by IL-1β may influence osteoclasts to induce bone loss (29). IL-1β promoted RANKL production by influencing immune cells (T-lymphocytes, B-lymphocytes, and macrophages), which subsequently bound to osteoclast precursor RANK, promoting osteoclast differentiation and activation. Thus, the inflammatory factor IL-1β produced by macrophages may modulate bone metabolism by impeding osteogenesis and facilitating osteoclasticity (30). IL-6 could similarly foster the differentiation of osteoclastic progenitor cells in this manner (31). IL-17A stimulated bone marrow mesenchymal stem cell (BMSC) secretion of RANKL and M-CSF, promoting osteoclastogenesis and augmenting bone resorption (32). In OVX mice, IL-18 stimulated the secretion of IL-17 by TH17 cells. This inhibited osteogenic differentiation and mineralization but promoted osteoclast differentiation and bone resorption. The administration of IL-18 antagonists alleviated osteoporosis in OVX mice (33). Hence, the immune microenvironment, comprising immune cells and inflammatory factors (IL-1β, IL-18, IL-17, IL-6, and TNF-α), plays a pivotal regulatory role in bone metabolism and contributes to the pathogenesis of osteoporosis (**Figure 1**).

## 3 The concept of pyroptosis and its correlated pathways

Inflammatory environments are recognized for their propensity to induce diverse modalities of cell death, encompassing apoptosis, autophagy, ferroptosis, necroptosis, and pyroptosis (34) (**Table 1**). Pyroptosis constitutes a variant of programmed cell death (PCD), characterized by genetically regulated, autonomous, and orchestrated cellular demise aimed at preserving bodily homeostasis (35). Pyroptosis is incited by an array of pathological stimuli, encompassing circumstances such as oxidative stress, hyperglycemia, and infection, and is presently observable across a diverse spectrum of cellular entities, including monocytes, macrophages, dendritic cells, osteoblasts, and BMSCs (36).

**Table 1:** Comparison between different forms of programmed cell death.

	Apoptosis	Autophagy	Ferroptosis	Necroptosis	pyroptosis
The triggering factor	Embryonic development, cell renewal, chemicals, oxidative stress, and radiation	Endogenous or exogenous sources of stress	Intracellular iron overload and ROS accumulation	Chemical and mechanical injuries, inflammation, or infection	Microbial infections, DAMPs, PAMPs
Inflammation-related	Anti-inflammatory	Partially have	Pro-inflammatory	pro-inflammatory	Pro-inflammatory
Key Factors	Bax, Bcl-2 family, caspase-2/3/6/7/8/9/10 and p53	Autolysosomes, mTOR and ATGs	SystemXc-/GSH/GPX4, GCH1/DHFR/BH4, FSP1/NADPH/CoQ10 and DHODH/CoQ10	RIPK1/RIPK3/MLKL	Caspase-1/3/4/5/8/11, Inflammasomes, GSDM protein family
Characteristics	Principal pathways encompass the mitochondrial pathway, the death receptor pathway, and the endoplasmic reticulum stress pathway; involving cellular matrix cleavage, nuclear condensation, DNA cleavage, and plasma membrane contraction, culminating in the generation of a conglomerate of apoptotic vesicles.	Genetically orchestrated spontaneous cell death, characterized by lysosomal phagocytosis and recycling of autophagic vesicles that encapsulate cytoplasmic contents for subsequent reuse.	Due to the accrual of toxic levels of free iron, depletion of the antioxidant glutathione (GSH), and oxidative damage to membrane lipids, intracellular lipid peroxidation is triggered, giving rise to the generation of detrimental lipid peroxides. These peroxides subsequently induce mitochondrial atrophy and outer membrane rupture.	The assembly of the necrosome, culminating in the breach of the plasma membrane, heightens intracellular osmotic pressure. This escalation leads to the seepage of cellular contents, organelle enlargement, mitochondrial dysfunction, and ultimately, the forceful rupture of the plasma membrane.	The principal pathways encompass the canonical pyroptosis pathway, non-canonical pyroptosis, and additional pathways activated by inflammasome and gasdermin family proteins. These proteins form pores across the cell membrane, releasing inflammatory factors and inducing cell swelling, rupture, and demise.
Cellular release	Apoptotic vesicles encapsulating cell debris	No extracellular product release	The massive release of oxidized lipid mediators and DAMPs(HMGB1)	Cell contents and DAMPs (IL-1α, IL-33, dsDNA and ATP, etc.)	Cell contents and inflammation factors like IL-18 and 1L-1β
Related diseases	Developmental disorders, autoimmune diseases, infectious diseases and cancer	Cancer, infectious diseases, metabolic diseases, cardiovascular and musculoskeletal diseases	Inflammatory diseases, tumors, and related conditions	Infectious, autoimmune, cardiovascular, gastrointestinal, and neoplastic diseases	Inflammatory diseases, infectious diseases, autoimmune disorders, neoplastic conditions, and related ailments

Apoptosis represents a meticulously regulated, inflammation-independent process of cellular demise instigated by factors like oxidative stress, radiation, and the loss of survival signals. It is triggered through the mitochondrial apoptosis pathway, the death receptor pathway, and the endoplasmic reticulum stress pathway (37, 38). This involves cleavage of the cell matrix, reduction in cell volume, chromatin condensation, nuclear fragmentation, and the wrapping of the plasma membrane around cellular debris to create apoptotic vesicles. Subsequently, these vesicles undergo phagocytosis and are removed for recycling by adjacent cells.

Autophagy stands as a pivotal pathway crucial for preserving homeostasis in cells and organisms. Induced by various stimuli, cells engulf damaged organelles, proteins, and other materials utilizing double-membrane autophagosomes, subsequently transporting them to the lysosome for recycling. Autophagy plays a pivotal role in the development, differentiation, and organizational homeostasis of organisms (39).

Ferroptosis epitomizes an iron-dependent mode of cell death wherein the accrual of free iron toxicity, depletion of the antioxidant glutathione (GSH), and oxidative damage to membrane lipids collectively initiate the generation of reactive oxygen species (ROS) from intracellular free iron via the Fenton reaction. This process involves the attack on polyunsaturated fatty acids in the cell membrane, culminating in the formation of detrimental lipid peroxides. The manifestation of ferroptosis is characterized by an intact cell membrane with atrophied mitochondria, reduced mitochondrial cristae, and rupture of the outer membrane (40).

Amidst environmental stress, encompassing cytochemical and mechanical injury, inflammation, or infection, the activation of TNF-α initiates the formation of complex I from RIPK1. In the absence of caspase-8, the interaction of RIPK1 and RIPK3 gives rise to the formation of complex IIb (necrosome). This necrosome is further triggered by the phosphorylation of mixed-lineage kinase domain-like protein (MLKL), resulting in the creation of a plasma membrane pore, ultimately inducing necroptosis (41).

**Figure 2:**
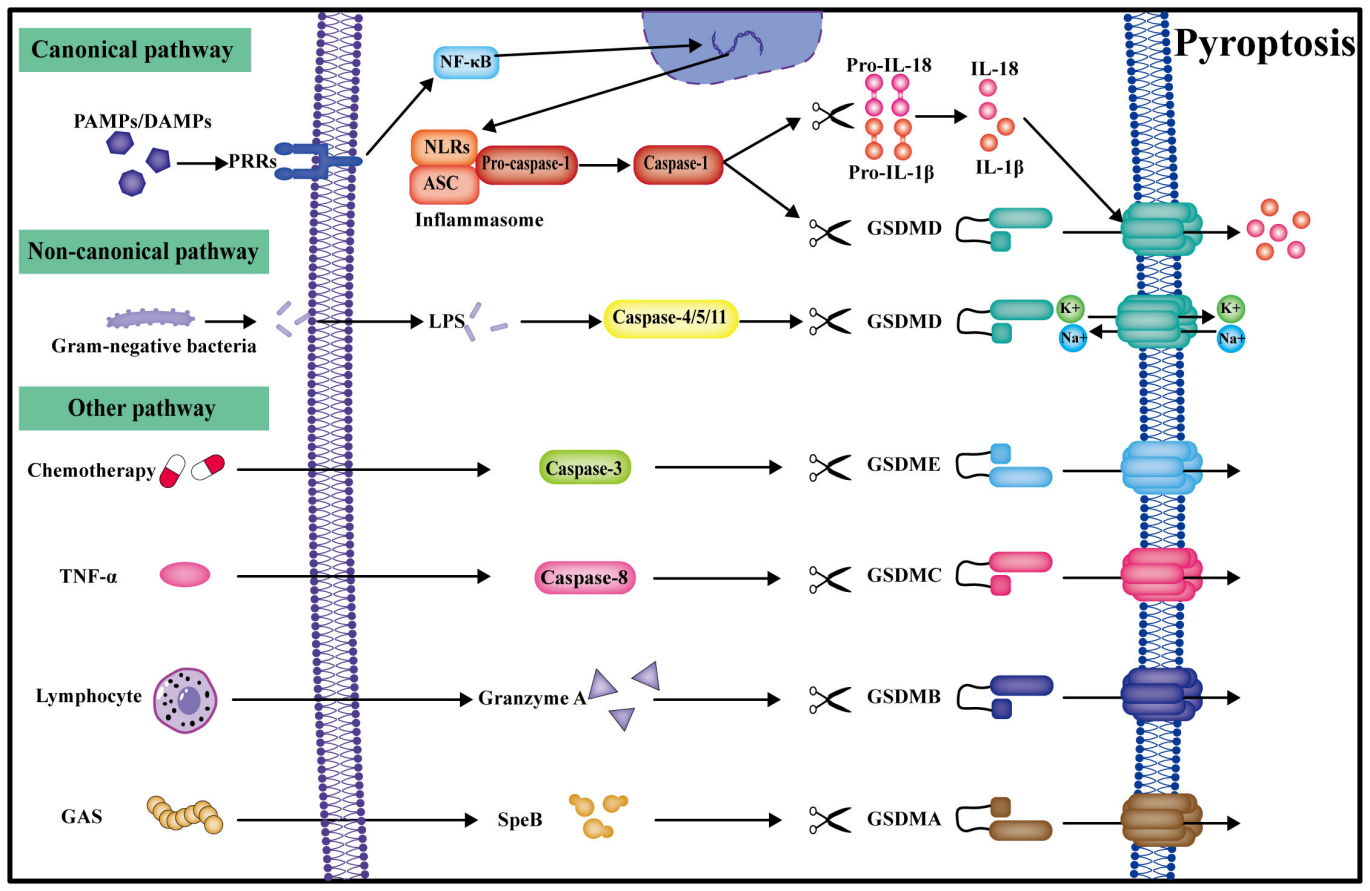
Pyroptosis can be categorized into canonical, non-canonical, and other pathways. In the canonical pathway, PRRs get activated by multiple signals, transduced by NF-κB signaling to form the inflammasome. Caspase-1 cleaves GSDMD to create the plasma membrane pore, and IL-18 and IL-1β are also cleaved by caspase-1 into active forms for release into the extracellular space, ultimately leading to pyroptosis. In the non-classical pathway, caspase-4/5/11 can be directly activated by LPS and undergo pyroptosis by directly cleaving GSDMD. Other gasdermin pyroptosis pathways mediated by caspase-3/8, granzyme A, and SpeB are also identified.

Pyroptosis, orchestrated by Gasdermin, represents a programmed cell death mechanism characterized by cell expansion culminating in cell membrane rupture and the subsequent release of cellular contents, further amplifying the inflammatory response (42). Described as the most inflammatory mode of death, pyroptosis can be categorized into canonical pyroptosis pathways, non-canonical pyroptosis pathways, and other GSDM-mediated pyroptosis pathways (**Figure 2**) (43).

### 3.1 Caspase-1-dependent canonical pyroptosis pathway

In the canonical pyroptosis pathway, pattern recognition receptors (PRRs) become activated by pathogen-associated molecular patterns (PAMPs), encompassing bacteria and viruses, and damage-associated molecular patterns (DAMPs), involving uric acid crystals, saturated fatty acids, and cholesterol (44). Inflammatory signals are transmitted through the intracellular NF-κB pathway, initiating the assembly of the inflammasome, which consolidates into the inflammasome complex within the cell. Key constituents of this complex comprise NLR proteins, such as NLRP1, NLRP3, NLRC4, NLRC5, PYD, and AIM2, among others (45). The inflammasome complex subsequently cleaves pro-caspase-1 into an enzymatically active form (46). Caspase-1 further processes gasdermin D (GSDMD), cleaving it into a water-soluble GSDMD C-terminal structural domain and a lipid-soluble GSDMD N-terminal structural domain (47). The N-terminal structural domain undergoes oligomerization, forming a permeable pore in the cell membrane. Due to the disparate osmotic pressures of sodium and potassium ions inside and outside the cell, leading to water influx into the cell through an osmotic pressure gradient, the cell swells, ruptures, and ultimately perishes (48). Concurrently, caspase-1 cleaves and matures the inflammatory factors IL-1β and IL-18 precursors, releasing them extracellularly through the GSDMD pore. Extracellular inflammatory factors can further recruit immune cells, amplifying the inflammatory response and inducing pyroptosis, thereby creating an inflammatory microenvironment (49).

### 3.2 Caspase4/5/11-dependent non-canonical pyroptosis pathway

Human caspase-4 and caspase-5 exhibit homology with rodent caspase-11, all of which express the CARD structural domain. Within the non-canonical pyroptosis pathway, lipopolysaccharide (LPS) derived from the cell wall of Gram-negative bacteria is directly recognized by the CARD structural domain in the cytoplasm, initiating pyroptosis with the involvement of IFN-γ. Intracellular LPS directly cleaves GSDMD via activated caspase-4/5/11, forming GSDMD pores in the cell membrane, ultimately leading to pyroptosis (50).

### 3.3 Other GSDM-mediated pyroptosis pathways

Other investigations have demonstrated that, following stimulation by chemotherapeutic drugs or TNF, caspase-3 could trigger pyroptosis by cleaving GSDME and generating membrane pores from GSDME fragments. Silencing GSDME in mice improved tissue damage and weight loss induced by chemotherapy, suggesting that caspase-3 may have mediated cell pyroptosis through the GSDME pathway (51). Another study disclosed that caspase-8 induced embryonic lethality in mice by triggering necroptosis and pyroptosis, showcasing caspase-8 as a molecular switch that governs apoptosis and pyroptosis while averting tissue damage during embryonic development and into adulthood (52). In cancer cells, PD-L1 could transform TNF-α-induced apoptosis into pyroptosis, involving caspase-8 and GSDMC (53). Lymphocytes might have anti-tumor effects by fostering pyroptosis of GSDMB-positive cells through granzyme A (GZMA) (54). Streptococcus pyogenes, also recognized as Group A Streptococcus (GAS), generated SpeB virulence factors that induced pyroptosis in skin keratinocytes by cleaving GSDMA (55).

## 4 Impact of pyroptosis on bone metabolism

### 4.1 Pyroptosis inhibits osteogenesis

Osteoblasts were differentiated from BMSCs, and a plethora of lipid droplets were observed in the marrow cavity of OVX-induced osteoporotic mice, indicating that lipogenic differentiation was much more pronounced than osteoprogenitor differentiation in OP. Pyroptosis has been demonstrated to actively participate in this process (56). The viability and osteogenic differentiation of BMSCs were diminished in the inflammatory environment, while the expression of NLRP3 and IL-1β in BMSCs significantly increased under LPS stimulation, suggesting that BMSCs pyroptosis inhibits osteogenic differentiation (57). A study has demonstrated that treatment of osteoblasts with sodium butyrate (NaB) induced pyroptosis and inhibited osteogenesis through the caspase-3/GSDME and OPG/RANKL axis, resulting in diminished osteogenic differentiation (58). Treatment of bone marrow stromal cells (ST2) with dexamethasone (DEX) induced pyroptosis and hindered osteogenic differentiation; this effect was notably alleviated by silencing the caspase-1 gene (59). The upregulation of NLRP3, caspase-1, ASC, and IL-1β expression in osteoblasts from OVX mice suggested that OVX inhibited osteogenesis by promoting osteoblast pyroptosis. Melatonin therapy attenuated this pathway and bolstered bone formation via the Wnt/β-catenin pathway (60). In the heterotopic ossification model, macrophage pyroptosis affected tendon stem cell senescence via IL-1β and HMGB1-containing extracellular vesicles, leading to the formation of heterotopic ossification, suggesting that macrophage pyroptosis played an important role in the regulation of bone metabolism (61). Upon stimulation with TNF-α, osteoblast MC3T3-E1 underwent pyroptosis, which is regulated by ELP2, a key protein in the JAK-STAT3 pathway. Additionally, the ELP2 protein inhibited osteogenic differentiation through activation of the NLRP3-GSDMD/GSDME pathway (62). Estrogen deficiency curtailed osteogenic differentiation of BMSCs by promoting the Capsase-1/GSDMD/IL-18 and IL-1β pyroptosis pathways, culminating in osteoporosis, while NLRP3 knockdown fostered bone formation (26). The above research suggests that pyroptosis is important in the regulation of osteogenesis, which may be inhibited by inducing osteoblast pyroptosis through either direct or indirect effects.

### 4.2 Pyroptosis promotes bone resorption

LPS-induced pyroptosis in bone marrow macrophages (BMMs), resulted in the secretion of the inflammatory factor IL-1β, which promoted osteoclast differentiation and increased bone resorption. However, osteoclast differentiation could be suppressed by inflammasome inhibitors (MCC950 and Z-YVAD-FMK) (63). Research has also indicated that the occurrence of pyroptosis in BMDMs might stimulate the secretion of IL-1 β, thereby promoting the differentiation and maturation of osteoclasts (64). Mimicking the human phenotype, NLRP3 mice^D301N type^ exhibited systemic inflammation and inflammatory bone loss due to increased bone resorption by osteoclasts via actin cytoskeleton reorganization (65). The expression of pyroptosis-related proteins was higher in osteomyelitis models than in uninfected bone, so inhibiting pyroptosis could alleviate the bone destruction due to osteomyelitis via attenuating the abnormal activation of osteoclasts (66). The NLRP3 inhibitor glyburide reduced the expression of pyroptosis-related proteins and reversed bone resorption in a periodontitis model by inhibiting pyroptosis (67). GSDMD limited lysosomal transport and maturation in osteoclasts, regulating their activity. In the OVX mouse model, bone loss was more severe in GSDMD knockout mice, indicating that pyroptosis regulates alterations in bone metabolism by GSDMD in osteoclasts (68).

**Figure 3:**
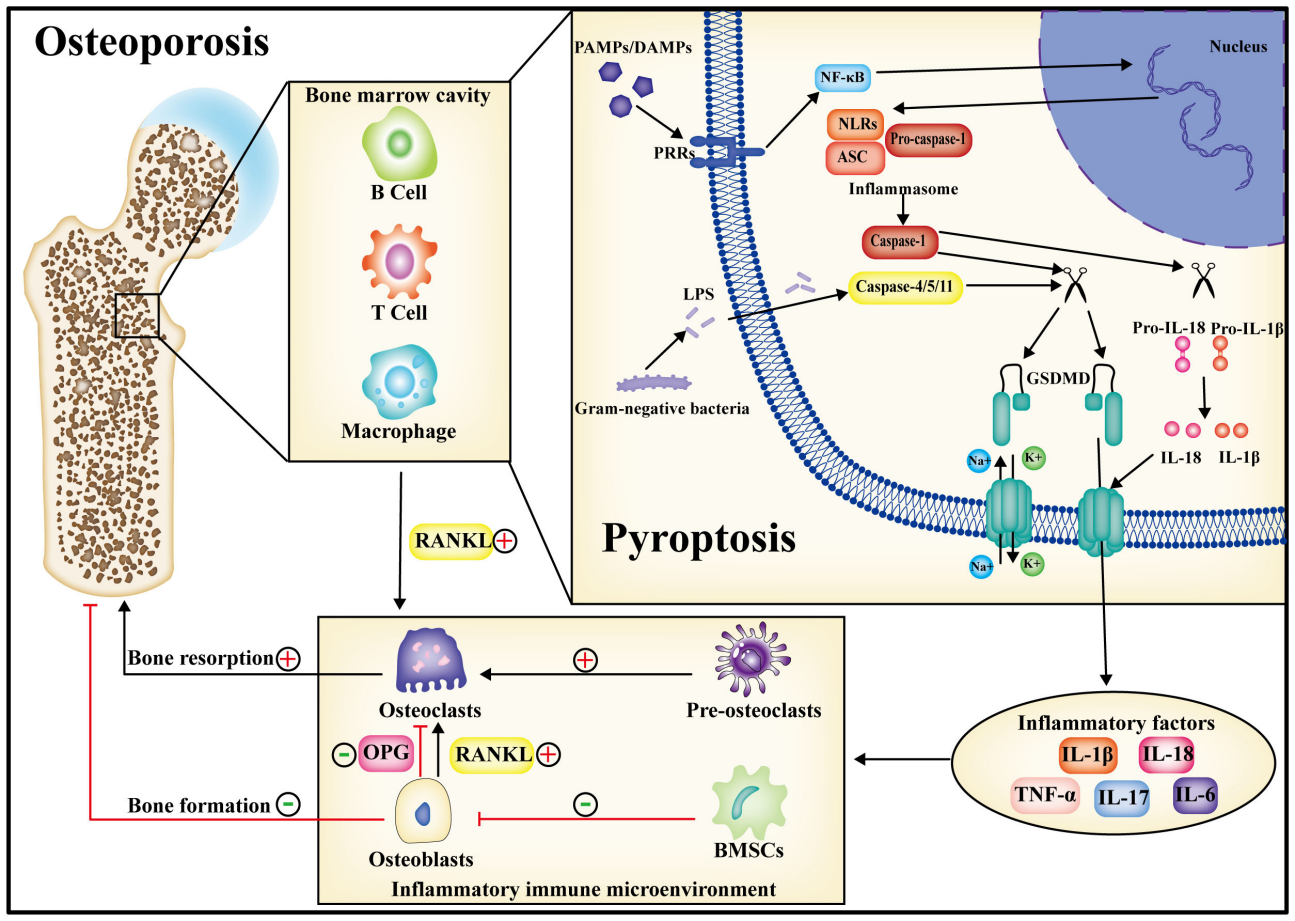
During the progression of osteoporosis, pyroptosis may occur in both immune cells and bone-metabolizing cells. Following pyroptosis, inflammatory factors are released into the bone marrow cavity, giving rise to a chronic inflammatory immune microenvironment. Within this microenvironment, the homeostasis of bone metabolism is perturbed, ultimately contributing to osteoporosis by amplifying bone resorption and diminishing bone synthesis. Consequently, the inhibition of pyroptosis is anticipated to emerge as a therapeutic target for the treatment of osteoporosis.

In summary, pyroptosis represents a form of innate immune death, and its advantages and disadvantages are contingent upon the duration of the pyroptotic process. In the initial phase of pyroptosis, cells release substantial amounts of inflammatory factors through this mechanism, and the rupture of the cell membrane exposes intracellular pathogens to antibodies, facilitating their phagocytosis and destruction by recruited macrophages. Nevertheless, in the presence of persistent pyroptosis mediated by caspase and GSDMD, there is an extensive secretion of inflammatory factors, including TNF-α, IL-1β, IL-18, and IL-6, etc, creating a chronic immune inflammatory microenvironment. In the context of this inflammatory immune microenvironment, immune cells persistently secreted RANKL, thereby intensifying the stimulation of macrophage osteoclastic differentiation and amplifying bone resorption. Simultaneously, the osteogenic differentiation capability of BMSCs was diminished, impeding osteoblast activity and undermining bone synthesis. Concurrently, the capacity of osteoblasts to secrete OPG was compromised, transmitting this effect to osteoclasts. Consequently, the signaling interplay between osteoblasts and osteoclasts became dysregulated, culminating in the disturbance of bone metabolic homeostasis and the manifestation of diverse aseptic and bacterial inflammatory bone disorders (**Figure 3**).

## 5 Pyroptosis has the potential to emerge as a novel therapeutic target for osteoporosis

Earlier studies have demonstrated that estrogen deficiency can induce the inflammatory immune microenvironment. Under the influence of this microenvironment, the balance of bone metabolism is disrupted, eventually leading to the development of osteoporosis. Consequently, interrupting this cycle to regulate the inflammatory immune microenvironment holds therapeutic potential for curing osteoporosis. Presently, osteoporosis is managed by inhibiting bone resorption and promoting bone formation. Commonly utilized anti-resorptive drugs include bisphosphonates, estrogen receptor modulators, and monoclonal antibodies targeting RANKL (69). Drugs that stimulate bone formation encompass teriparatide (recombinant human parathyroid hormone analog - rPTH), vitamin D, vitamin K, and other small-molecule supplements (70). Both direct inhibitors and indirect drugs targeting pyroptosis are available. The subsequent section outlines drugs specifically designed to address pyroptosis in the context of osteoporosis (**Table 2**).

**Table 2:** Drugs targeting pyroptosis in the context of osteoporosis.

Products	Targets	Treatment effects	References
Urolithin A	osteoclast(NF-κB/NLRP3/Caspase-1/GSDMD)	Administration of Urolithin A inhibited osteoclast-mediated bone resorption by modulating the NF-ΚB/NLRP3/Caspase-1/GSDMD pathway.	(71)
Rosmarinic acid	osteoclast(NLRP3)	Rosmarinic acid has the potential to alleviate streptozotocin-induced diabetic osteoporosis by suppressing NLRP3 in osteoclasts.	(72)
Irisin	BMSCs(NLRP3)	Irisin inhibited NLRP3-mediated pyroptosis in BMSCs and alleviated streptozotocin-induced osteoporosis.	(73)
Melatonin	Osteoblast(NLRP3)	Melatonin alleviated osteoporosis by suppressing NLRP3 activation through the Wnt/β-catenin pathway.	(60)
Anemarrhenae Rhizoma/Phellodendri Chinensis Cortex (AR/PCC)	Osteoblast(NLRP3)	AR/PCC alleviated diabetes-related osteoporosis by upregulating the antioxidant response protein (Nrf2) to reduce the activation of the NLRP3-mediated canonical pyroptosis pathway in vertebral osteoblasts.	(74)
MCC950	osteoclast(NLRP3)	The NLRP3 inhibitor MCC950 mitigated age-related bone loss by inhibiting osteoclast differentiation, thereby reducing osteoclastic effects, in line with the observations in NLRP3 knockout mice.	(75)
Dioscin	Macrophage(NLRP3、Caspase-1)	Diosgenin inhibited Enterococcus faecalis-induced macrophage pyroptosis and enhanced osteogenic differentiation of MC3T3-E1 cells.	(76)
AC-YVAD-CMK	Osteoblast(Caspase-1)	Under high glucose conditions, MC3T3-E1 cells underwent pyroptosis through the caspase-1/GSDMD/IL-1β axis, and the caspase-1 inhibitor AC-YVAD-CMK augmented osteoblast proliferation and differentiation functions.	(77)

Urolithin A (UA) is a biologically active metabolite produced by the gut microbiota, exhibiting beneficial effects on cell function (78). *In vivo*, studies demonstrated that 8 weeks of UA gavage increased bone mass in OVX mice, with UA achieving bone mass restoration by inhibiting RANKL-driven osteoclast activation. Sequencing analysis indicated a potential correlation with the reduction in inflammatory factor expression mediated by UA. Conversely, *in vitro* findings revealed that UA can regulate macrophage polarization and attenuate inflammatory responses. UA inhibits osteoclastogenesis by reducing pyroptosis through the inhibition of NLRP3, suggesting that the strategy of inhibiting pyroptosis-induced inflammation by UA may be valuable for PMOP therapy (71).

Rosmarinic acid (RA) is a naturally occurring phenolic acid compound isolated from plants, displaying potent antioxidant activity. Clinical investigations have demonstrated its benefits in dermatological, allergic, and osteoarthritic diseases, as well as in improving cognitive performance and treating metabolic syndrome (79). RA was found to ameliorate streptozotocin (STZ)-induced diabetic osteoporosis in rats by inhibiting the activation of the inflammasome NLRP3 in osteoclasts. This suggests that RA may play a role in the treatment of diabetic osteoporosis (72).

Irisin, a hydrolysis product of fibronectin type III domain-containing 5 (FNDC5), undergoes endoplasmic reticulum processing, facilitating its secretion into the bloodstream to intricately regulate energy metabolism. Irisin levels exhibit a robust correlation with health status, revealing markedly diminished levels in individuals afflicted with obesity, osteoporosis, reduced bone mass, muscular dystrophy, and cardiovascular disease, while cancer patients manifest elevated irisin levels (80). A study conducted *in vivo* demonstrated that an 8-week running regimen led to elevated irisin levels in mice, potentially enhancing bone mineral density in OVX mice (81). Subsequent investigations revealed that the promotion of elevated irisin levels via exercise and *in vivo* administration mitigated the signaling pathways associated with inflammasome-induced pyroptosis in diabetic mice, consequently impeding bone loss. This implies a potential therapeutic role of irisin in alleviating osteoporosis through the inhibition of pyroptosis (73).

Melatonin, an indoleamine primarily secreted by the pineal gland, exhibits a distinct circadian rhythm. Numerous studies have elucidated its capacity to exert antioxidative, anti-inflammatory, anti-tumorigenic, and anti-aging functions (82). The deduction that melatonin possesses the capability to counteract the advancement of osteoarthritis and is employed to forestall cartilage deformation while stimulating cartilage matrix synthesis underscores its advantageous impact on the intricacies of bone metabolism (83). Observations revealed that intraperitoneal administration of melatonin for 8 weeks mitigated osteoporosis induced by OVX in a dosage-dependent manner. Additionally, *in vitro*, treatment of BMSCs with melatonin facilitated osteogenic differentiation, with this effect attributed to the inhibition of NLRP3 inflammasome activation through the Wnt/β-catenin pathway. This demonstrates melatonin’s potential to ameliorate estrogen deficiency-induced osteoporosis by suppressing pyroptosis in BMSCs (60).

Anemarrhenae Rhizoma/Phellodendri Chinensis Cortex (AR/PCC), a distinct herbal combination extensively utilized in Traditional Chinese Medicine (TCM) for managing diabetes, has demonstrated a profound therapeutic impact in addressing diabetic osteoporosis. Administering AR/PCC orally alleviated streptozotocin-induced diabetic osteoporosis in a rat model. Additionally, AR/PCC demonstrated blood glucose reduction, upregulation of osteoblast-related genes, and downregulation of osteoclast-related genes in a diabetic zebrafish model (84). Subsequent *in vivo* immunofluorescence analyses unveiled that AR/PCC mitigated STZ-induced diabetic osteoporosis in rats by suppressing osteoblast pyroptosis through activation of the Nrf2/Keap1 pathway (74).

MCC950, an NLRP3-selective inhibitor, exerts its inhibitory effect on inflammasome activation by suppressing ASC oligomerization and binding to NLRP3. This compound has demonstrated therapeutic efficacy in animal models of autoimmune, cardiovascular, metabolic, and other diseases (85). Earlier studies proposed that MCC950 inhibits NLRP3 activation and diminishes the production of the inflammatory factor IL-1β in macrophages (86). *In vivo* and *in vitro* experiments demonstrated that MCC950 alleviates osteoarthritis by suppressing inflammation and safeguarding cartilage (87). Furthermore, MCC950 treatment effectively mitigated age-related bone loss by inhibiting osteoclast activation, aligning with findings in NLRP3 knockout mice. These results suggest that MCC950 inhibits NLRP3 inflammasome activation in osteoclasts, thereby mitigating bone loss (87).

Dioscin, a naturally occurring bioactive steroidal saponin, exhibits protective effects against a spectrum of ailments, including malignant tumors, metabolic disorders, organ damage, and infectious diseases in humans. It is extensively utilized in Chinese medicine (88). Observations indicate that Dioscin inhibits macrophage pyroptosis induced by Enterococcus faecalis and stimulates osteogenic differentiation of MC3T3-E1 cells *in vitro*. These findings suggest the potential utility of dioscin in promoting osteogenesis, particularly in combating osteoporosis (76).

AC-YVAD-CMK, a selective caspase-1 inhibitor, demonstrates efficacy in significantly diminishing the expression of IL-18 and IL-1β. Inhibiting synovial macrophage pyroptosis with Ac-YVAD-CMK was observed to mitigate synovitis and fibrosis in a rat model of knee osteoarthritis (89). Subsequent investigations revealed that administering the inhibitors Ac-YVAD-CMK and MCC050, targeting caspase-1 and NLRP3, respectively, effectively alleviated pyroptosis in synovial tissues of temporomandibular joint osteoarthritis (90). Under high glycemic conditions, MC3T3-E1 cells underwent pyroptosis through the caspase-1/GSDMD/IL-1β axis. The caspase-1 inhibitor AC-YVAD-CMK was shown to enhance osteoblast proliferation and differentiation (77).

## 6 Conclusion and future perspectives

Osteoporosis progression frequently coincides with inflammation, wherein pyroptosis presently stands recognized as one of the most inflammatory modalities of cell death. Immune cells, along with their associated counterparts, elicit pyroptosis via canonical and non-canonical pyroptosis pathways, resulting in the secretion of inflammatory factors, including IL-18, IL-1β, TNF-α, and IL-6. This process establishes an inflammatory immune microenvironment. Through various signaling pathways, these inflammatory factors intricately regulate bone metabolism, culminating in an imbalance characterized by heightened bone resorption and diminished bone formation, ultimately resulting in osteoporosis. Previous investigations have substantiated that suppressing pyroptosis can mitigate the advancement of osteoporosis through anti-inflammatory actions. The current research on pyroptosis and osteoporosis still has many limitations. Although *in vitro* and animal models have successfully alleviated osteoporosis by inhibiting pyroptosis to ameliorate inflammatory bone loss, the precise mechanisms and specific regulatory pathways remain incompletely elucidated, and potential drug side effects require further clarification. Consequently, the hypothesis proposing that pyroptosis mediates osteoporosis via the inflammatory immune microenvironment necessitates additional, more comprehensive research to identify suitable targets and pharmaceutical interventions for osteoporosis treatment. We believe that osteoporosis development may be induced by estrogen deficiency in macrophages through pyroptosis, which may lead to the secretion of inflammatory factors such as IL-1β, IL-6, and TNF-α and the formation of an inflammatory immune microenvironment, under which conditions osteogenic activity is reduced and osteoclasts are enhanced, leading to an imbalance in bone metabolism and ultimately osteoporosis. Further research is required to clarify the specific signaling pathways upstream and downstream of pyroptosis, particularly in-depth analysis of its endpoint, the Gasdermin protein. It is recommended that new pyroptosis-inhibiting drugs be developed to investigate their effects on osteoporosis and to reduce drug side effects. Advanced research on inflammation-regulated osteoporosis to illustrate the mechanism of interaction between immune cells and bone metabolizing cells in the inflammatory microenvironment, including the direction of regulation.

## Author contributions

TC: Data curation, Writing – original draft, Writing – review & editing, Conceptualization, Supervision. LJ: Data curation, Supervision, Writing – original draft. JL: Conceptualization, Visualization, Writing – review & editing. YL: Supervision, Writing – review & editing.

## Glossary

**Table:**

PMOP	Postmenopausal osteoporosis
SOP	Senile osteoporosis
IOP	Idiopathic osteoporosis
WHO	World health organization
BMD	Bone mineral density
BMSCs	Bone marrow mesenchymal stem cells
DNA	Deoxyribonucleic acid
IL-1	Interleukin-1
IL-6	Interleukin-6
IL-17	Interleukin-17
TNF-α	Tumor necrosis factor
OPG	Osteoprotegerin
RANKL	Receptor activator of nuclear factor-κB ligand
M-CSF	Macrophage colony stimulating factor
AP-1	Activator protein 1
NF-κB	Nuclear factor -κB
Erα	Estrogen receptor-α
BCAR1	Breast cancer anti-estrogen resistance protein-1
BMP-4	Bone morphogenetic protein 4
SMAD	Drosophila mothers against decapentaplegic protein
PTH	Parathyroid hormone
OVX	Ovariectomy
NLRP3	NOD-like receptor family pyrin domain-containing 3
TH-17	T helper cell 17
IL-18	Interleukin-18
IL-1β	Interleukin-1β
PCD	programmed cell death
Bcl-2	B-cell lymphoma-2;Bax, Bcl-2 associated X protein
Caspase	Cysteine-aspartic protease
mTOR	mammalian target of rapamycin
ATGs	Autophagy-related proteins
GSH	Glutathione
GPX4	Glutathione peroxidase 4
GCH1	GTP cyclohydrolase 1
DHFR	Dihydrofolate reductase
BH4	Tetrahydrobiopterin
FSP1	Ferroptosis suppressor protein 1
NADPH	Nicotinamide adenine dinucleotide phosphate
CoQ10	Coenzyme Q10
DHODH	Dihydroorotate dehydrogenase
RIPK	Receptor-interacting protein kinase
MLKL	Mixed-lineage kinase domain-like protein
GSDM	Gasdermin
HMGB1	High-mobility group box 1 protein
DAMPs	Damage‐associated molecular patterns
PAMPs	Pathogen-related molecular patterns
ROS	Reactive oxygen species
PRRs	Pattern recognition receptors
NLR	NOD-like receptor
NLRP1	NLR family pyrin domain-containing 1
NLRC4	NLR family CARD domain containing 4
NRRC5	NLR family CARD domain containing 5
PYD	Pyrindomain
AIM2	Absent in melanoma 2
LPS	Lipopolysaccharide
PD-L1	Programmed death ligand 1
GZM	Granzyme
GAS	Group A Streptococcus
ASC	Apoptosis-related speck-like protein
BMMs	Bone marrow macrophages
rPTH	recombinant human parathyroid hormone analog
UA	Urolithin A
RA	Rosmarinic acid
STZ	streptozotocin
FNDC5	Fibronectin type III domain-containing 5
TCM	Traditional Chinese Medicine
Nrf2	Nuclear factor erythroid 2-related factor 2
Keap1	Kelch-like ECH-associated protein-1.

## References

[1] Akkawi I Zmerly H. Osteoporosis: current concepts. Joints. (2018) 6:122–7. doi: 10.1055/s-0038-1660790 PMC6059859 30051110

[2] Sözen T Özışık L Başaran NÇ. An overview and management of osteoporosis. Eur J Rheumatol. (2017) 4:46–56. doi: 10.5152/eurjrheumatol. 28293453 PMC5335887

[3] Wright NC Looker AC Saag KG Curtis JR Delzell ES Randall S. The recent prevalence of osteoporosis and low bone mass in the United States based on bone mineral density at the femoral neck or lumbar spine. J Bone Miner Res. (2014) 29:2520–6. doi: 10.1002/jbmr.2269 PMC4757905 24771492

[4] Kok C Sambrook PN. Secondary osteoporosis in patients with an osteoporotic fracture. Best Pract Res Clin Rheumatol. (2009) 23:769–79. doi: 10.1016/j.berh.2009.09.006 19945688

[5] Srivastava RK Sapra L Mishra PK. Osteometabolism: metabolic alterations in bone pathologies. Cells-Basel. (2022) 11. doi: 10.3390/cells11233943 PMC9735555 36497201

[6] Rao Z Zhu Y Yang P Chen Z Xia Y Qiao C. Pyroptosis in inflammatory diseases and cancer. Theranostics. (2022) 12:4310–29. doi: 10.7150/thno.71086 PMC9169370 35673561

[7] Almeida M Laurent MR Dubois V Claessens F O'Brien CA Bouillon R. Estrogens and androgens in skeletal physiology and pathophysiology. Physiol Rev. (2017) 97:135–87. doi: 10.1152/physrev.00033.2015 PMC5539371 27807202

[8] Camacho PM Petak SM Binkley N Diab DL Eldeiry LS Farooki A. AMERICAN ASSOCIATION OF CLINICAL ENDOCRINOLOGISTS/AMERICAN COLLEGE OF ENDOCRINOLOGY CLINICAL PRACTICE GUIDELINES FOR THE DIAGNOSIS AND TREATMENT OF POSTMENOPAUSAL OSTEOPOROSIS-2020 UPDATE. Endocr Pract. (2020) 26:1–46. doi: 10.4158/GL-2020-0524SUPPL 32427503

[9] Datta HK Ng WF Walker JA Tuck SP Varanasi SS. The cell biology of bone metabolism. J Clin Pathol. (2008) 61:577–87. doi: 10.1136/jcp.2007.048868 18441154

[10] Aarshageetha P Janci P Tharani ND. Role of alternate therapies to improve the quality of life in menopausal women: A systematic review. J Midlife Health. (2023) 14:153–8. doi: 10.4103/jmh.jmh_222_22 PMC10836436 38312763

[11] Duursma SA Raymakers JA Boereboom FT Scheven BA. Estrogen and bone metabolism. Obstet Gynecol Surv. (1992) 47:38–44. doi: 10.1097/00006254-199201000-00015 1734333

[12] Nilsson S Mäkelä S Treuter E Tujague M Thomsen J Andersson G. Mechanisms of estrogen action. Physiol Rev. (2001) 81:1535–65. doi: 10.1152/physrev.2001.81.4.1535 11581496

[13] Bajpai AK Gu Q Jiao Y Starlard-Davenport A Gu W Quarles LD. Systems genetics and bioinformatics analyses using ESR1-correlated genes identify potential candidates underlying female bone development. Genomics. (2024) 116:110769. doi: 10.1016/j.ygeno.2023.110769 38141931 PMC10811775

[14] Alake SE Ice J Robinson K Price P Hatter B Wozniak K. Reduced estrogen signaling contributes to bone loss and cardiac dysfunction in interleukin-10 knockout mice. Physiol Rep. (2024) 12:e15914. doi: 10.14814/phy2.15914 38217044 PMC10787104

[15] Shevde NK Bendixen AC Dienger KM Pike JW. Estrogens suppress RANK ligand-induced osteoclast differentiation via a stromal cell independent mechanism involving c-Jun repression. Proc Natl Acad Sci U.S.A. (2000) 97:7829–34. doi: 10.1073/pnas.130200197 PMC16630 10869427

[16] Robinson LJ Yaroslavskiy BB Griswold RD Zadorozny EV Guo L Tourkova IL. Estrogen inhibits RANKL-stimulated osteoclastic differentiation of human monocytes through estrogen and RANKL-regulated interaction of estrogen receptor-alpha with BCAR1 and Traf6. Exp Cell Res. (2009) 315:1287–301. doi: 10.1016/j.yexcr.2009.01.014 PMC2765696 19331827

[17] Gavali S Gupta MK Daswani B Wani MR Sirdeshmukh R Khatkhatay MI. Estrogen enhances human osteoblast survival and function via promotion of autophagy. Biochim Biophys Acta Mol Cell Res. (2019) 1866:1498–507. doi: 10.1016/j.bbamcr.2019.06.014 31255720

[18] Matsumoto Y Otsuka F Takano-Narazaki M Katsuyama T Nakamura E Tsukamoto N. Estrogen facilitates osteoblast differentiation by upregulating bone morphogenetic protein-4 signaling. Steroids. (2013) 78:513–20. doi: 10.1016/j.steroids.2013.02.011 23499826

[19] Almeida M Iyer S Martin-Millan M Bartell SM Han L Ambrogini E. Estrogen receptor-α signaling in osteoblast progenitors stimulates cortical bone accrual. J Clin Invest. (2013) 123:394–404. doi: 10.1172/JCI65910 23221342 PMC3533305

[20] Schiavi J Fodera DM Brennan MA McNamara LM. Estrogen depletion alters osteogenic differentiation and matrix production by osteoblasts in *vitro* . Exp Cell Res. (2021) 408:112814. doi: 10.1016/j.yexcr.2021.112814 34492267

[21] Agnusdei D Civitelli R Camporeale A Gennari C. Calcitonin and estrogens. J Endocrinol Invest. (1990) 13:625–30. doi: 10.1007/BF03349583 2273204

[22] Naveh-Many T Almogi G Livni N Silver J. Estrogen receptors and biologic response in rat parathyroid tissue and C cells. J Clin Invest. (1992) 90:2434–8. doi: 10.1172/JCI116134 PMC443399 1469095

[23] Weitzmann MN Pacifici R. Estrogen regulation of immune cell bone interactions. Ann N Y Acad Sci. (2006) 1068:256–74. doi: 10.1196/annals.1346.030 16831927

[24] Fischer V Haffner-Luntzer M. Interaction between bone and immune cells: Implications for postmenopausal osteoporosis. Semin Cell Dev Biol. (2022) 123:14–21. doi: 10.1016/j.semcdb.2021.05.014 34024716

[25] Srivastava RK Dar HY Mishra PK. Immunoporosis: immunology of osteoporosis-role of T cells. Front Immunol. (2018) 9:657. doi: 10.3389/fimmu.2018.00657 29675022 PMC5895643

[26] Yang C Song B Han L Gao Z. Study on the mechanism of NLRP3 effect on the skeleton of de-ovalized mice. Biochem Biophys Rep. (2023) 35:101496. doi: 10.1016/j.bbrep.2023.101496 37332667 PMC10276222

[27] Hirano S Zhou Q Furuyama A Kanno S. Differential regulation of IL-1β and IL-6 release in murine macrophages. Inflammation. (2017) 40:1933–43. doi: 10.1007/s10753-017-0634-1 28766178

[28] Chien SY Tsai CH Liu SC Huang CC Lin TH Yang YZ. Noggin inhibits IL-1β and BMP-2 expression, and attenuates cartilage degeneration and subchondral bone destruction in experimental osteoarthritis. Cells-Basel. (2020) 9. doi: 10.3390/cells9040927 PMC7226847 32290085

[29] Simsa-Maziel S Zaretsky J Reich A Koren Y Shahar R Monsonego-Ornan E. IL-1RI participates in normal growth plate development and bone modeling. Am J Physiol Endocrinol Metab. (2013) 305:E15–21. doi: 10.1152/ajpendo.00335.2012 23592480

[30] Ruscitti P Cipriani P Carubbi F Liakouli V Zazzeroni F Di Benedetto P. The role of IL-1β in the bone loss during rheumatic diseases. Mediators Inflammation. (2015) 2015:782382. doi: 10.1155/2015/782382 PMC4410538 25954061

[31] Wang T He C. TNF-α and IL-6: the link between immune and bone system. Curr Drug Targets. (2020) 21:213–27. doi: 10.2174/1389450120666190821161259 31433756

[32] Tang M Lu L Yu X. Interleukin-17A interweaves the skeletal and immune systems. Front Immunol. (2020) 11:625034. doi: 10.3389/fimmu.2020.625034 33613566 PMC7890031

[33] Mansoori MN Shukla P Kakaji M Tyagi AM Srivastava K Shukla M. IL-18BP is decreased in osteoporotic women: Prevents Inflammasome mediated IL-18 activation and reduces Th17 differentiation. Sci Rep. (2016) 6:33680. doi: 10.1038/srep33680 27649785 PMC5030484

[34] Jorgensen I Rayamajhi M Miao EA. Programmed cell death as a defence against infection. Nat Rev Immunol. (2017) 17:151–64. doi: 10.1038/nri.2016.147 PMC5328506 28138137

[35] Bedoui S Herold MJ Strasser A. Emerging connectivity of programmed cell death pathways and its physiological implications. Nat Rev Mol Cell Bio. (2020) 21:678–95. doi: 10.1038/s41580-020-0270-8 32873928

[36] Awad F Assrawi E Louvrier C Jumeau C Georgin-Lavialle S Grateau G. Inflammasome biology, molecular pathology and therapeutic implications. Pharmacol Ther. (2018) 187:133–49. doi: 10.1016/j.pharmthera.2018.02.011 29466702

[37] Cory S Adams JM. The Bcl2 family: regulators of the cellular life-or-death switch. Nat Rev Cancer. (2002) 2:647–56. doi: 10.1038/nrc883 12209154

[38] Du C Fang M Li Y Li L Wang X. Smac, a mitochondrial protein that promotes cytochrome c-dependent caspase activation by eliminating IAP inhibition. Cell. (2000) 102:33–42. doi: 10.1016/S0092-8674(00)00008-8 10929711

[39] Miller DR Thorburn A. Autophagy and organelle homeostasis in cancer. Dev Cell. (2021) 56:906–18. doi: 10.1016/j.devcel.2021.02.010 PMC8026727 33689692

[40] Xu H Ye D Ren M Zhang H Bi F. Ferroptosis in the tumor microenvironment: perspectives for immunotherapy. Trends Mol Med. (2021) 27:856–67. doi: 10.1016/j.molmed.2021.06.014 34312075

[41] Khoury MK Gupta K Franco SR Liu B. Necroptosis in the pathophysiology of disease. Am J Pathol. (2020) 190:272–85. doi: 10.1016/j.ajpath.2019.10.012 PMC6983729 31783008

[42] Galluzzi L Vitale I Aaronson SA Abrams JM Adam D Agostinis P. Molecular mechanisms of cell death: recommendations of the Nomenclature Committee on Cell Death 2018. Cell Death Differ. (2018) 25:486–541. doi: 10.1038/s41418-018-0102-y 29362479 PMC5864239

[43] Du T Gao J Li P Wang Y Qi Q Liu X. Pyroptosis, metabolism, and tumor immune microenvironment. Clin Transl Med. (2021) 11:e492. doi: 10.1002/ctm2.492 34459122 PMC8329701

[44] Jiang D Chen S Sun R Zhang X Wang D. The NLRP3 inflammasome: Role in metabolic disorders and regulation by metabolic pathways. Cancer Lett. (2018) 419:8–19. doi: 10.1016/j.canlet.2018.01.034 29339210

[45] Place DE Kanneganti TD. Recent advances in inflammasome biology. Curr Opin Immunol. (2018) 50:32–8. doi: 10.1016/j.coi.2017.10.011 PMC5857399 29128729

[46] Broz P Dixit VM. Inflammasomes: mechanism of assembly, regulation and signalling. Nat Rev Immunol. (2016) 16:407–20. doi: 10.1038/nri.2016.58 27291964

[47] Shi J Zhao Y Wang K Shi X Wang Y Huang H. Cleavage of GSDMD by inflammatory caspases determines pyroptotic cell death. Nature. (2015) 526:660–5. doi: 10.1038/nature15514 26375003

[48] Sborgi L Rühl S Mulvihill E Pipercevic J Heilig R Stahlberg H. GSDMD membrane pore formation constitutes the mechanism of pyroptotic cell death. EMBO J. (2016) 35:1766–78. doi: 10.15252/embj.201694696 PMC5010048 27418190

[49] Ding J Wang K Liu W She Y Sun Q Shi J. Pore-forming activity and structural autoinhibition of the gasdermin family. Nature. (2016) 535:111–6. doi: 10.1038/nature18590 27281216

[50] Yang J Zhao Y Shao F. Non-canonical activation of inflammatory caspases by cytosolic LPS in innate immunity. Curr Opin Immunol. (2015) 32:78–83. doi: 10.1016/j.coi.2015.01.007 25621708

[51] Wang Y Gao W Shi X Ding J Liu W He H. Chemotherapy drugs induce pyroptosis through caspase-3 cleavage of a gasdermin. Nature. (2017) 547:99–103. doi: 10.1038/nature22393 28459430

[52] Newton K Wickliffe KE Maltzman A Dugger DL Reja R Zhang Y. Activity of caspase-8 determines plasticity between cell death pathways. Nature. (2019) 575:679–82. doi: 10.1038/s41586-019-1752-8 31723262

[53] Hou J Zhao R Xia W Chang CW You Y Hsu JM. PD-L1-mediated gasdermin C expression switches apoptosis to pyroptosis in cancer cells and facilitates tumour necrosis. Nat Cell Biol. (2020) 22:1264–75. doi: 10.1038/s41556-020-0575-z PMC7653546 32929201

[54] Zhou Z He H Wang K Shi X Wang Y Su Y. Granzyme A from cytotoxic lymphocytes cleaves GSDMB to trigger pyroptosis in target cells. Science. (2020) 368. doi: 10.1126/science.aaz7548 32299851

[55] Deng W Bai Y Deng F Pan Y Mei S Zheng Z. Streptococcal pyrogenic exotoxin B cleaves GSDMA and triggers pyroptosis. NATURE. (2022) 602:496–502. doi: 10.1038/s41586-021-04384-4 35110732 PMC9703647

[56] Wang L Chen K Wan X Wang F Guo Z Mo Z. NLRP3 inflammasome activation in mesenchymal stem cells inhibits osteogenic differentiation and enhances adipogenic differentiation. Biochem Biophys Res Commun. (2017) 484:871–7. doi: 10.1016/j.bbrc.2017.02.007 28167279

[57] Wang X Jiang M He X Zhang B Peng W Guo L. N−acetyl cysteine inhibits the lipopolysaccharide−induced inflammatory response in bone marrow mesenchymal stem cells by suppressing the TXNIP/NLRP3/IL−1β signaling pathway. Mol Med Rep. (2020) 22:3299–306. doi: 10.3892/mmr PMC7453581 32945495

[58] Wu Z Ding Q Yue M Zhang X Han D Zhang L. Caspase-3/GSDME-mediated pyroptosis leads to osteogenic dysfunction of osteoblast-like cells. Oral Dis. (2023). doi: 10.1111/odi.14579 37004144

[59] Ruan H Zhang H Feng J Luo H Fu F Yao S. Inhibition of Caspase-1-mediated pyroptosis promotes osteogenic differentiation, offering a therapeutic target for osteoporosis. Int Immunopharmacol. (2023) 124:110901. doi: 10.1016/j.intimp.2023.110901 37839278

[60] Xu L Zhang L Wang Z Li C Li S Li L. Melatonin suppresses estrogen deficiency-induced osteoporosis and promotes osteoblastogenesis by inactivating the NLRP3 inflammasome. Calcif Tissue Int. (2018) 103:400–10. doi: 10.1007/s00223-018-0428-y 29804160

[61] Li J Wang X Yao Z Yuan F Liu H Sun Z. NLRP3-dependent crosstalk between pyroptotic macrophage and senescent cell orchestrates trauma-induced heterotopic ossification during aberrant wound healing. Adv Sci (Weinh). (2023) 10:e2207383. doi: 10.1002/advs.202207383 37204068 PMC10323626

[62] Xia C Ou S Yang Y Zhang W Wu W Chen Q. ELP2-NLRP3-GSDMD/GSDME-mediated pyroptosis is induced by TNF-α in MC3T3-E1 cells during osteogenic differentiation. J Cell Mol Med. (2023) 27:4093–106. doi: 10.1111/jcmm.17994 PMC10746952 37830762

[63] Alam MI Mae M Farhana F Oohira M Yamashita Y Ozaki Y. NLRP3 inflammasome negatively regulates RANKL-induced osteoclastogenesis of mouse bone marrow macrophages but positively regulates it in the presence of lipopolysaccharides. Int J Mol Sci. (2022) 23. doi: 10.3390/ijms23116096 PMC9181162 35682777

[64] Wu YL Zhang CH Teng Y Pan Y Liu NC Liu PX. Propionate and butyrate attenuate macrophage pyroptosis and osteoclastogenesis induced by CoCrMo alloy particles. Mil Med Res. (2022) 9:46. doi: 10.1186/s40779-022-00404-0 35996168 PMC9396885

[65] Qu C Bonar SL Hickman-Brecks CL Abu-Amer S McGeough MD Peña CA. NLRP3 mediates osteolysis through inflammation-dependent and -independent mechanisms. FASEB J. (2015) 29:1269–79. doi: 10.1096/fj.14-264804 PMC4396608 25477279

[66] Zhu X Zhang K Lu K Shi T Shen S Chen X. Inhibition of pyroptosis attenuates Staphylococcus aureus-induced bone injury in traumatic osteomyelitis. Ann Transl Med. (2019) 7:170. doi: 10.21037/atm 31168451 PMC6526268

[67] Jiang M Shang Z Zhang T Yin X Liang X Sun H. Study on the role of pyroptosis in bone resorption induced by occlusal trauma with or without periodontitis. J Periodontal Res. (2022) 57:448–60. doi: 10.1111/jre.12974 35141913

[68] Li M Yang D Yan H Tang Z Jiang D Zhang J. Gasdermin D maintains bone mass by rewiring the endo-lysosomal pathway of osteoclastic bone resorption. Dev Cell. (2022) 57:2365–80. doi: 10.1016/j.devcel.2022.09.013 36243012

[69] De Martinis M Sirufo MM Ginaldi L. Osteoporosis: current and emerging therapies targeted to immunological checkpoints. Curr Med Chem. (2020) 27:6356–72. doi: 10.2174/0929867326666190730113123 PMC8206194 31362684

[70] Wang H Luo Y Wang H Li F Yu F Ye L. Mechanistic advances in osteoporosis and anti-osteoporosis therapies. MedComm (2020). (2023) 4:e244. doi: 10.1002/mco2.244 37188325 PMC10175743

[71] Tao H Li W Zhang W Yang C Zhang C Liang X. Urolithin A suppresses RANKL-induced osteoclastogenesis and postmenopausal osteoporosis by, suppresses inflammation and downstream NF-κB activated pyroptosis pathways. Pharmacol Res. (2021) 174:105967. doi: 10.1016/j.phrs.2021.105967 34740817

[72] Li Q Tao X Zhang Y. Rosmarinic acid alleviates diabetic osteoporosis by suppressing the activation of NLRP3 inflammasome in rats. Physiol Int. (2022). doi: 10.1556/2060.2022.00154 35230263

[73] Behera J Ison J Voor MJ Tyagi N. Exercise-linked skeletal irisin ameliorates diabetes-associated osteoporosis by inhibiting the oxidative damage-dependent miR-150-FNDC5/pyroptosis axis. DIABETES. (2022) 71:2777–92. doi: 10.2337/db21-0573 PMC9750954 35802043

[74] Fu F Luo H Du Y Chen Y Tian K Pan J. AR/PCC herb pair inhibits osteoblast pyroptosis to alleviate diabetes-related osteoporosis by activating Nrf2/Keap1 pathway. J Cell Mol Med. (2023) 27:3601–13. doi: 10.1111/jcmm.17928 PMC10660633 37621124

[75] Zang Y Song JH Oh SH Kim JW Lee MN Piao X. Targeting NLRP3 inflammasome reduces age-related experimental alveolar bone loss. J Dent Res. (2020) 99:1287–95. doi: 10.1177/0022034520933533 32531176

[76] Yin W Liu S Dong M Liu Q Shi C Bai H. A new NLRP3 inflammasome inhibitor, dioscin, promotes osteogenesis. SMALL. (2020) 16:e1905977. doi: 10.1002/smll.201905977 31814281

[77] Yang L Liu J Shan Q Geng G Shao P. High glucose inhibits proliferation and differentiation of osteoblast in alveolar bone by inducing pyroptosis. Biochem Biophys Res Commun. (2020) 522:471–8. doi: 10.1016/j.bbrc.2019.11.080 31780258

[78] D'Amico D Andreux PA Valdés P Singh A Rinsch C Auwerx J. Impact of the natural compound urolithin A on health, disease, and aging. Trends Mol Med. (2021) 27:687–99. doi: 10.1016/j.molmed.2021.04.009 34030963

[79] Hitl M Kladar N Gavarić N Božin B. Rosmarinic acid-human pharmacokinetics and health benefits. Planta Med. (2021) 87:273–82. doi: 10.1055/a-1301-8648 33285594

[80] Liu S Cui F Ning K Wang Z Fu P Wang D. Role of irisin in physiology and pathology. Front Endocrinol (Lausanne). (2022) 13:962968. doi: 10.3389/fendo.2022.962968 36225200 PMC9549367

[81] Kawao N Iemura S Kawaguchi M Mizukami Y Takafuji Y Kaji H. Role of irisin in effects of chronic exercise on muscle and bone in ovariectomized mice. J Bone MINER Metab. (2021) 39:547–57. doi: 10.1007/s00774-020-01201-2 33566209

[82] Reiter RJ Tan DX Galano A. Melatonin: exceeding expectations. Physiol (Bethesda). (2014) 29:325–33. doi: 10.1152/physiol.00011.2014 25180262

[83] Zhang Y Liu T Yang H He F Zhu X. Melatonin: A novel candidate for the treatment of osteoarthritis. Ageing Res Rev. (2022) 78:101635. doi: 10.1016/j.arr.2022.101635 35483626

[84] Xu P Lin B Deng X He S Chen N Wang N. Anti-osteoporosis effects of Anemarrhenae Rhizoma / Phellodendri Chinensis Cortex herb pair and its major active components in diabetic rats and zebrafish. J Ethnopharmacol. (2022) 293:115269. doi: 10.1016/j.jep.2022.115269 35398497

[85] Li H Guan Y Liang B Ding P Hou X Wei W. Therapeutic potential of MCC950, a specific inhibitor of NLRP3 inflammasome. Eur J Pharmacol. (2022) 928:175091. doi: 10.1016/j.ejphar.2022.175091 35714692

[86] Coll RC Robertson AA Chae JJ Higgins SC Muñoz-Planillo R Inserra MC. A small-molecule inhibitor of the NLRP3 inflammasome for the treatment of inflammatory diseases. Nat Med. (2015) 21:248–55. doi: 10.1038/nm.3806 PMC4392179 25686105

[87] Ni B Pei W Qu Y Zhang R Chu X Wang Y. MCC950, the NLRP3 inhibitor, protects against cartilage degradation in a mouse model of osteoarthritis. Oxid Med Cell Longev. (2021) 2021:4139048. doi: 10.1155/2021/4139048 34777685 PMC8580635

[88] Bandopadhyay S Anand U Gadekar VS Jha NK Gupta PK Behl T. Dioscin: A review on pharmacological properties and therapeutic values. Biofactors. (2022) 48:22–55. doi: 10.1002/biof.1815 34919768

[89] Zhang L Xing R Huang Z Zhang N Zhang L Li X. Inhibition of synovial macrophage pyroptosis alleviates synovitis and fibrosis in knee osteoarthritis. Mediators Inflammation. (2019) 2019:2165918. doi: 10.1155/2019/2165918 PMC6754937 31582897

[90] Xin Y Wang W Mao E Yang H Li S. Targeting NLRP3 inflammasome alleviates synovitis by reducing pyroptosis in rats with experimental temporomandibular joint osteoarthritis. Mediators Inflammation. (2022) 2022:2581151. doi: 10.1155/2022/2581151 PMC9712023 36466156

